# Combating insolvency and business recovery problems in the oil industry: proposal for improvement in Nigeria's insolvency and bankruptcy legal framework

**DOI:** 10.1016/j.heliyon.2021.e06123

**Published:** 2021-02-11

**Authors:** Olusola Joshua Olujobi

**Affiliations:** Elizade University, Faculty of Law, Department of Public and International Law, Ilara -Mokin, Ondo State, Nigeria

**Keywords:** Nigeria, Oil firms, Creditor, Insolvency and business recovery laws

## Abstract

Insolvency and business recovery laws in Nigeria have not evolved to incorporate reorganisation, reforming insolvent oil firms' operations to boost commercial oil firms' steadiness and economic suitability like other moderately developed countries. In Nigeria, liquidation is understood by many as the panacea to indebtedness. The research evaluates the Nigerian insolvency and business recovery legal regime to sustain indebted oil firms from economic shocks due to the global decline in the oil price to avert imminent business failures due to insufficient cash flows. The aim is to fill the gaps in Nigeria's insolvency and business recovery laws by recommending a model for the sustenance of oil firms and to suggest the reform of the gaps identified in the existing laws and the extant literature on the subject. The paper opted for conceptual legal review, comparative legal and policies analyses of solvency and business recovery legislations in Nigeria, Malaysia, India, South Africa, the United Kingdom and the United States. These nations were designated for this study because their insolvency and business recovery legal regime are business rescued driven, not winding up centred. The study is library research-based to address some of the flaws in Nigeria's insolvency and business recovery laws. The study finds that Nigerian legislation on insolvency is flawed in oil firms' salvage, improvement and rearrangement. It ends that, statutory bodies in the designated case study nations are efficient than those in Nigeria due to the strong political will of their governments in supporting insolvent oil firms for successful financial recovery, to safeguard jobs, to protect creditors and to enhance the wealth of their nations through sound business recovery policies and laws. The study, advocates, remodel of Nigeria's insolvency and business recovery legislations and policies in compliance with the international standards on insolvent oil firms salvaged and creditors focused policies for a robust economy. The study concludes with the recommendation for further study to consider quantitative analysis research methodology to project further scholarship on the subject.

## 1 Introduction

Globally, the call to overhaul insolvency and business recovery laws have been a concern to many nations, as many countries are now making efforts to enhance their insolvency laws and practices to focus on companies salvage and lenders focused on enhancing fiscal stability, business propriety and on averting loss of means of livelihoods. The remodel was done in the designated nations to improve their insolvency and business recovery procedures that Nigeria can replicate to boost its business recovery laws due to the current global economic shocks occasioned by the pandemic which had affected global economic activities negatively.

Insolvency is the failure of an oil firm to fulfil its financial obligations (Rashid Shamim 2019). Section 572(a)(b)(c) of the Companies and Allied Matters Act, 2020 and Section 408(d) of Bankruptcy and Insolvency (Repeal and Re**-**enactment) Act, 2016, describes insolvency as the failure of oil firms to recompense financial obligations particularly lenders by an assignment which the firm is owed a sum above 2,000.00. The lender must give notification of its claims to the firm at its head office demanding the sum which is outstanding for three (3) weeks and after the expiration of the ultimatum where such oil firm fails or declined to disburse the unpaid amount to the approval of the lender (Adebola, 2012), such an oil firm is deemed to be insolvent.

This situation often arises where the court made a directive in the interest of a creditor of an oil firm. The directive is not obeyed; the court of law can deliberate on the oil firm's dependent obligations. Where insolvency occurs, the firm may, by order of the court, be declared insolvent by petition brought by the creditors, the debtors or by an act of agreement with the creditors, which its assets should be sold to settle the debt as soon as the assets can be disbursed to offset the debts (Chepkemoi Too, 2015).

Generally, many upstream oil firms are now closing their oil business. Some are leaving Nigeria due to debts, unsafe market restructuring strategies, a slump in global oil markets prices and a lack of funds to finance strategic oil ventures. Chevron, Total, Royal Dutch Shell and Eni, among others, have disbursed several of their onshore possessions in the sector due to governing policies changes. There is a need for the Federal Government to intervene to prevent job losses due to post COVID -19 adverse effects.

The Bankruptcy Act relates to insolvency proceedings against individual debtors and associations on its own. At the same time, the Companies and Allied Matters Act 2020, regulates the termination of oil firms and arrangement and compromises. The latter choice is not often utilised in practice since most insolvent lawyers tend to compel the courts to shut down and terminate the life of insolvent oil firms devoid of allowing the debtor firms to restructure its operations and repay its debts over time. Though winding up is the final alternative in other advanced countries, it is seen as the first preference in some African countries, leading to the untimely death of many oil firms that could have been recovered if they had a chance to handle restructures their debts properly (Fatula, 2007).

The aim is to modernise insolvency law by overhauling the legal regime regulating corporate law practice in Nigeria since insolvency of oil firms’ occasioned shocks, resentment, among oil firms, investors, regulatory authorities and other stakeholders in the industry (Frisby, 2011). Transformation of insolvency laws and practice will reduce conflicts between creditors, insolvent oil firms and combat impending insolvency signs at the initial stage among oil firms.

Furthermore, there is the need to realise the assets of the insolvent oil firms legitimately by discharging the debts promptly, allocate the proceeds realised from the sales of the insolvent's oil firm's assets amongst the creditors in an unbiased modus, refunding the excess to the debtors (insolvent oil firms). The rationale for this is to protect sustainable oil firms capable of adding up to Nigeria's economy by restoring their financial viability and workforce to boost investors' confidence in the industry (Olujobi et al., 2018).

Therefore, the proposed model's focus on insolvency law is to encourage the prospect of reforming insolvent oil firms and their enterprises to ensure the continuity of business that will result in long-term jobs, disbursement of levies and dividends and additional socio-economic advantages to Nigerians. Therefore, in line with the global trend of nipping insolvency in the bud, there is a need for an inclusive transformation of the extant insolvency law by encouraging mechanisms for business rescue and restructuring in the oil industry since the rationale of the law of bankruptcy is to collect the debt of an oil firm and allocate the assets among the creditors (Adegbemi et al., 2017). It will resolve business failure problems and folded up of oil firms to facilitate speedy recovery of debts and preserve employment (Menezes, and Muro, 2020).

In the global economic crisis, the desire for liquidity usually intensifies the danger of pushing viable oil firms into winding up particularly small oil firms. Therefore, they occasioned employment losses and the sale of assets of indebted or insolvent oil firms.

The goal of this analysis is to perform comparative legal and policy analyses of insolvency and business recovery laws in Nigeria, Malaysia, India, South Africa, the United Kingdom and the United States in order to update Nigeria's insolvency and business recovery laws and policies, from the winding-up of insolvent oil firms to the reorganisation, consolidation and to overhaul the laws and operations of those oil firms to promote economic stability in the country to ensure sound oil firms are giving a fair chance to survive the expected temporary market disruption pending the economic stability of the sector via debt restructuring mechanisms.

The study is apportioned into five parts. Section one includes the overview, section two addresses the methodology, the statement of the problem, the theoretical structure, the national regulatory mechanism for insolvency and an oil firm recovery in Nigeria, section three, defines the administrative, legislative environment and analyses of the existing legal framework of Nigeria, Malaysia, India and South Africa, the United Kingdom and the United States. Section 4 discusses the signs to look out whether oil firm's business is in insolvency and section five addresses turnaround or redemption plans for insolvent oil firms and other solutions available to unsecured creditors with the various realistic practices strategies Nigeria should learn from the chosen case study nations. The paper concluded with recommendations and policy implication of the study.

## 2 Methodology

This analysis's key objective is to explore how the nucleus of insolvency and oil firm business recovery activities in Nigeria will change from prosecution of insolvent oil firms by liquidation to reorganisation, consolidation of those oil firms to improve economic stability, financial property and industrial growth. To accomplish this aim, the author utilised bibliography-based doctrinal legal research methods which are primarily library-based, accompanied by analysis and a guide from internet references, a systematic examination of scholarly literature, a review of case studies and an overview of the applicable legal and regulatory stipulations. The research is based on secondary sources, such as periodicals, textbooks, and primary sources, such as judicial precedents and statutory provisions with comparative legal and policy analyses of insolvency and business recovery laws in Nigeria, Malaysia India, South Africa, United Kingdom and the United States. The research proposes using the lessons learnt to reform the gaps identified in Nigeria's legal regime.

## 3 Statement of the problem

In Nigeria, most oil firms in acute financial crises often end up being liquidated, which is harmful to the economy due to employment losses. However, some of these oil firms can be profitable and can be restructured for the good of their borrowers, debtors, and Nigeria's economy. Some ailing state**-**owned oil firms pre-insolvency may raise capital by selling their shares to the public for sale through an initial public offering or private placement or the stock market, as elaborated in Section 2 of the Public Sector (Privatisation and Commercialization) Act, 1999.

Some private oil firms can also raise capital by borrowing from financial firms or via other suitable means, rather than liquidating or winding up those oil firms businesses in Nigeria. Therefore, there is a need for a paradigm shift to recover financially distressed oil firms by recovering their properties and liabilities and conducting negotiations with their creditors to reach a definitive consensus on the repayment plan of the loan on agreed stipulated time by the parties (Asada, and Olong, 2007).

### 3.1 Theoretical framework on insolvency and business recovery practices

Donaldson and Davis introduced the philosophy of stewardship in 1991. The philosophy centred on the idea that the needs of shareholders and the desires of management should be matched and directed to take actions to the best advantage of the oil firm's success and its intrinsic values (Donaldson, and Davis, 1991).

The philosophy argues that there is more importance in joint activities for the firm's performance than individual interests. In contrast, the board's actions, the regulatory bodies, should be directed at increasing the resources of the owners (shareholders) and equally satisfying their desires. The board and the management of the oil firms are obligated to make the most of its owners' resources by investing, selling and reforming by doing so, optimising the assets and opportunities of the insolvent oil firm.

To achieve this aim, the government must reform the regulatory system governing insolvency and corporate recovery procedures for business-rescue, reconstruction and consolidation strategies for job-savings, which are guided by creditors’ strategies for a stable economy for the country. Management activities of oil firms are better encouraged where the corporate governance systems grant them strong authority and power to ensure that the firm stays in operation effectively to represent the needs of the shareholders, to improve the economy of the nation and to support the interests of all stakeholders in the field Donaldson and Davis (1991) recognised five modules of organisation management: honesty, transparent collaboration, leadership, long-term outlook, market success, and economic development, which will boost the profitability and sustainability of the oil firm.

In comparison, the second principle applicable to this analysis is the resource dependence theory (Olujobi, Olujobi, 2020) Pfeffer and Salancik founded it in 1978 with the goal of emphasising the importance of the role played by all stakeholders, such as the government regulatory bodies, the board of directors and shareholders, in improving the efficiency of the oil industry and preventing it from liquidation. Oil firms need capital for investment in crude oil exploration activities, inestimable human resources department roles, easily accessible scientific data, communique and skill to work correctly to attain oil firms’ corporate aims.

Generally, it is argued that resource accessibility improves the service, profitability, efficiency and market longevity of oil firms. Some researchers have suggested that the hypothesis reflects the vital role that government's policymakers and managers play in supplying the firm with critical services via their connexions to the outside world. They claim that managements add capital to the firm with data, expertise to get vendors, customers, public policymakers, social groups and credibility of their products.

The principle focuses on the means of access to capital for the activities of oil producers vital to their progress. According to (Pfeffer and Salancik 1978) the boards' roles include guidance, advice and professional know-how, credibility and prestige, a conduit for transmitting knowledge to other agencies, and exclusive access to agreements or funding from crucial players. The boards undertake these roles through social and technical networking. Zahra and Pearce (1989) suggest that the varied history of the managements increases the consistency of their recommendations to the oil business, thus improving the firm's viability and survival without insolvency.

Another good idea is that utilitarianism's ethical philosophy, which is based on the fairness and wrongness of actions; this relies solely on the truth of optimising human well-being as a whole. It is based on the highest benefit for the largest number. It encourages individuals to behave in some manners that can result in the most significant possible amount of well-being for the general population: David Hume (1979) Argued in his philosophical essays that much of our moral thought is mostly dictated by what we consider reasonable. The emphasis of utilitarianism on well-being is deeply rooted in human nature. Jeremy Bentham (2010) claimed that, as the total amount of enjoyment is raised, ethics can be adequately upheld worldwide for good conduct. This will enhance efficiency in the oil industry.

### 3.2 National legal regimes on insolvency and business recovery practices

The critical legislative mechanism for personal insolvency, otherwise known as bankruptcy in the country, is the Bankruptcy Act Cap. B2 LFN, 2004 and the Rules of Procedure laid down in the Bankruptcy Rules B2, LFN, 2004. The Federal High Court Rules augment the law following the terms of the Bankruptcy Act. The Bankruptcy and Insolvency Act 2016 and the Bankruptcy Laws govern insolvency proceedings in Nigeria. The Companies and Allied Matters Act, 2020, Investment and Securities Act, 2007 (No. 29), Investment and Securities Rules, 2013, Banks and Other Financial Institutions Act, Cap. B3, LFN, 2004 (as amended), Sections 16 and 21 of the Nigeria Deposit Insurance Corporations Act Failed Banks (Recovery of Debts and Financial Malpractices in Banks) Act, Cap F2, LFN 2004, Secured Transactions of in Moveable Assets Act, 2017 No.3, A43, and Central Bank of Nigeria Act, Cap. C4, LFN, 2010, among others are the cardinal national legal framework regulating insolvency in Nigeria.

Insolvency and corporate restructuring practice can be practiced by any person but exclude anyone under the age of 21 years, incompetent persons, individuals who have been declared bankrupt by a court of competent jurisdiction and individuals who have been sentenced by the court. Insolvency rule affects businesses that cannot settle their financial obligations, while bankruptcy refers to persons and partnership businesses in Nigeria.

Moreover, section 108 of the Bankruptcy Act (as amended) by Decree No. 109 of the 1992 Cap. B2 LFN 2004 forbids the issuance of an injunction under the Act against any firm or corporation listed in the Companies and Allied Matters Act 2020. The process for winding up insolvent oil firms is governed primarily by the winding-up proceedings under the Companies and Allied Matters Act, 2020. Section 7 of the Federal High Court Act, Chapter F12, LFN, 2004 and Section 251(1)(e) of the 1999 Constitution of the Federal Republic of Nigeria (as amended) allowed the Federal High Court to deal with insolvency cases. The claimant's place of business rather than the place of residence is considered for instituting insolvency proceedings in court against the insolvent oil firm (Ibiyemi, 1999). Restitution of debts by debtors to creditors can be taken before the Federal High Court, based on the jurisdiction where the individuals have executed the arrangements and subject to each case's circumstances.

On the other hand, winding-up processes commence by lodging a petition in the Federal High Court under section 578(1) Companies Allied Matters Act 2020. Where the claimant carries on business for a more significant part of the six months immediately before the filing of the petition, even though the petitioner no longer remains in that judicial division, but he is entitled to appeal in the Court of Appeal and lastly at the Supreme Court as stated under section 570(1) Companies Allied Matters Act 2020.

A borrower may file a bankruptcy petition against a debtor who owes more than One Million Naira 1,000,000.00 or is bankrupt within (6) six months of filing the petition. Investment and Securities Act, 2007 and Securities and Exchange Commission Regulations, 2013 control mergers, acquisitions and purchases of equity of listed public oil firms in the country.

However, another odd feature about the insolvency profession is that corporate bodies are not permitted to join the practice by statute. A private organisation cannot be a trustee under bankruptcy section 123 of the Bankruptcy Act, Cap. B2, LFN, 2004. Section 627 Companies and Allied Matters Act 2020 provide for a liquidator, but a corporate entity cannot be named a receiver or liquidator under the Act.

Besides, the Banks and Other Financial Institutions Act (BOFIA), Chapter B3, LFN, 2004 governs the consolidation, rearrangement, amalgamations, and financial institution transfer. Section 7 of the Banks and other Financial Institutions Act bans the consolidation, reorganisation, acquisition, and disposition of any gain or benefit in financial entity devoid of the Governor of the Central Bank of Nigeria's assent. The Nigeria Deposit Insurance Company Act, Cap N, 102, LFN, 2004 governs the protection of savings liabilities of approved banks and other financial institutions to cover the benefit of investors in the case of inevitable fiscal problems.

Sections 658 and 659, made a substantial amendment on the repealed section 495, CAMA 2004. The section declares as void unjustifiable or unfair creditor preference and authorises the court to reinstate properties sold lower value or devalued. Section 658 CAMA 2020 prohibits the company from any act which may bestow undue preference on any creditor as illegal. Also, section 658 proscribe any sale by the firm two years or three months before the insolvency to connected or unconnected individuals this may be declared invalid by the liquidator. Connected persons are employees, directors and close relatives. Section 659 of CAMA 2020 enables the court to annul undervalued contracts executed by the firm.

The following legal deficiencies are found, among others, in the Nigerian insolvency legal system, there are the absence of a well**-**organised process for managing debts of insolvent oil firms, lack of comprehensive and specific insolvency and firm rehabilitation regulations in Nigeria's legal regime to avoid the liquidation of potentially viable oil firms and poorly managed oil firms, owing to insolvency litigations by the congested courts and vulnerability to bribes, leading to considerable delays and obstruction of justice in the hearing of insolvency proceedings in courts in the country (Leonard, 2017).

### 3.3 Institutional frameworks regulating insolvency and business recovery practices in Nigeria

Section 7(1)(c) of the Banks and other Financial Institutions Act, Cap. B3 LFN, 2004 stipulates that without the prior approval of the Governor of the Central Bank of Nigeria, no financial institution shall implement any agreement or arrangement with any other person for the merger or amalgamation of such financial institution.

Nigeria's Central Bank has a function to perform by ensuring that no financial institution in Nigeria concludes any merger or amalgamation agreement with another bank without its approval and failure attracts a fine not less than 1,000,000 and where there is an ongoing wrongdoing an extra penalty of 10,000 for every day the breach persists. The Banks and Other Financial Institutions Act, Section 7(2), Cap. B LFN, 2004. The statutory roles of the Central Bank of Nigeria and the Nigeria Deposit Insurance Corporation complement each other; their roles in the handling of failed or insolvent financial institutions. The Assets Management Corporation of Nigeria (Amendment) Act, 2015 is to resolve banks' non**-**performing loan and assets effectively (Soludo, 2004).

The Securities and Exchange Commission (SEC) is authorised to regulate investment and bond**-**related matters in Nigeria. It evaluates and supports the amalgamations and acquirements of oil firms. Its enabling legislation, the Investments and Securities Act, 2007 should be revised to incorporate an insolvent oil firms rescue system to ensure job security and enhance Nigeria's economy.

The Corporate Affairs Commission (CAC) is instituted under section 1(1) of the Companies Allied Matters Act 2020 to manage the Nigerian company registry, to investigate the activities of any oil firm to protect the interests and benefits of investors and the society, to regulate, supervise the establishment, registration, controlling and liquidation of oil firms in Nigeria in order to prevent fraud, irregularities and mismanagement (Asada, and Olong, 2007).

Section 119(1) of the Investments and Securities Act, 2007 describes a merger as a combination of the activities or any part of the responsibility or the interest of two or several firms or some of the activities of one or more firms and one or more of the entities of the undertaking.

According to Section 849 of CAMA, a merger may be made in any way, comprising acquisition or lease of the stocks, profit or possessions of the entity concerns, or by merger or other mixture with the other company concerns. The Federal High Court has exclusive powers to wind-up oil firms, as set out in Section 570(1) of the Companies and Allied Matters Act 2020. However, special courts supervised by judges who are experts in insolvency and bankruptcy with staff experts in this field of law are needed for efficiency in oil firm business rescue and recovery strategies (Olujobi, and Olusola-Olujobi, 2019).

Nigeria Deposit Insurance Corporation created under the Nigeria Deposit Insurance Corporation Act, Cap N, 102, LFN, 2004 controls the indemnification of payments liabilities of licensed banks and other financial institutions to protect depositors' interest the occasion of banks' economic intricacies. Recovery and Insolvency Practitioners’ Association of Nigeria, an association of insolvency experts with knowledge of business recovery and insolvency in Nigeria. It will ensure that its members retain such knowledge and perform their insolvency and business recovery work by their professional ethics in accordance with sections 704, 705(1)(2),706 CAMA 2020. The National Assembly should introduce a bill prohibiting uncertified persons' services as insolvent practitioners to entrench sound governance and professionalism in insolvency practice in Nigeria.

It must have a framework on the minimum prerequisites for practising as an insolvency expert with an up-to-date register of its members with continuing vocational training, the development of its members with the Code of Conduct and the discipline of its erring members, and there is a need for licensing of insolvent and business recovery practitioners in Nigeria. (Khan et al., 2014).

There is also the need for institutional reforms and cooperation with the statutory bodies that regulate insolvency practitioners in Nigeria with the supervisory bodies in the petroleum sector to reduce winding**-**up of oil firms on account of insolvencies. The Ministry of Petroleum and Energy Resources, supervised by the Ministry of Petroleum, is legally authorised to develop rules governing the oil sector through the Department of Petroleum Resources (DPR) to endorse oil exploration rules and refining in the country. The Ministry needs to do more to prevent the continuing failure of oil firms in the country due to insolvency and poor corporate governance (Olujobi, 2017).

#### 3.3.1 Comparison of municipal legal regime of Nigeria, Malaysia, India, South Africa, the United Kingdom and the United States on insolvency and business recovery laws

Several trends in business rescue and acceptable practices have emerged over the years. Many countries have revised and implemented their laws by these trends to have an adequate legal framework that incorporates good international practice on creditor rights and insolvency, as laid down by the World Bank and the United Nations Commission on International Trade Law (UNICITRAL).

In England and Wales Insolvency Act, 1986 (as amended) governs insolvency while the Directors Disqualification Act, 1986 governs insolvent companies' directors. The insolvency law of England and Wales does not define insolvency, but Nigeria, Bankruptcy and Insolvency (Repeal and Re-enactment) Act 2016 section 408(d) of defines insolvency as an inability to pay a debt that arises where the creditor, by assignment or otherwise, is liable to a sum of more than 2,000 to the firm when the registered office is applying for payment of the outstanding amount if, after the expiration of three weeks, the firm refused to pay the outstanding amount to the reasonable satisfaction of the creditor. However, in other selected case study countries, the Act only defines the inability to pay as a failure to pay a claim due to a creditor exceeding the amount of £750 within three weeks after the written notice of request has been given (Opara et al., 2014).

England and Wales have no superior insolvency court, but their High Court can close any firm registered in England and Wales. The United Kingdom Insolvency Act 1986 section 240(2), define clearly relevant time and connected person as a director, shadow director or an acquaintance of such a director.

However, in the United States insolvency cases are dealt with by bankruptcy courts, a division of the Federal District Courts with limited jurisdiction. In contrast, in Nigeria, no higher court has been designated as insolvency court and is being held solely by the Federal High Court, as provided for in section 251(1)(e) of the 1999 Constitution of the Federal Republic of Nigeria (as amended).

In Malaysia, Section 176 of the Companies Act, 1965 established specialised courts to handle insolvency proceedings with provisions for ou**t-**of**-**court workouts, allowing creditors to participate in decision-making, applying strict rules insolvency practitioners to prevent fraud and unethical insolvency practices. Malaysia has also been practising some of these acceptable business rescue procedures, and this has proved to be an effective way to resolve insolvency cases faster and efficiently (Douglas -Abubakar, 2018).

The Dodd-Frank Wall Street Reform and Consumer Protection Act, 2010 and the Insolvent Partnerships Order, 1994 (as amended) regulate the insolvency of partnerships in the United States, the Bankruptcy Abuse Prevention and Consumer Protection Act (2005) transformed individual bankruptcy procedure in the United States. Chapter 13 bankruptcy law necessitates debtors to streamline their debts and to initiate three or five years repayment plan; it allows the debtor utilise future revenues to settle creditors wholly or partly (Kagan 2018).

In India, the Insolvency and Bankruptcy Code, 2016 regulates oil firms, partnerships and individuals. It helps firms make restructuring plans to pay up their debts and remain in business by protecting the interests of the parties by modifying the orderliness of payment of the debt and government charges. Insolvency Resolution Process empowers creditors to evaluate the insolvent oil firm's business viability whether to rescue it or liquidate it via a request to the National Company Law Tribunal. An insolvency professional may be appointed after the approval of the board. The Code makes provisions for speedy insolvency resolution procedure. The Code is very comprehensible and supportive of the insolvent oil firm and creditors. The Sick Industrial Companies (Special Provisions) Act, 1985, and Securitization and Reconstruction of Financial Assets and Enforcement of Security Interest Act, 2002 (April et al., 2019).

Insolvency in Malaysia is regulated by the Companies Act, 1965 and the Company Winding-Up Rules. In India, the Parliament adopted a second amendment to restructure the 1956 Insolvency Act, leading to a new corporate-business regime that was rescued in December 2002. Even though bankruptcies and winding-ups are very similar; the provisions dealing with them are contained in separate legislation, unlike some of the selected case study countries (Idigbe, 2011).

Similarly, the South Africa Companies Act 2008 restructured its arrangement and compromised the provisions of the law eliminate one phase of insolvency proceedings which required a court application before a meeting. In 2016, the United Kingdom's Government reformed its insolvency laws to incorporate restructuring and rearrangement.

In other relatively advanced climes where the insolvency laws have been judiciously restructured, for instance, the United Kingdom and the United States, the operations of the insolvent oil firm may persist under an acknowledged protection procedure. Preference recovery options are being formulated between the parties. The protection plan may offer, duration, for the debtor firms to have their properties under the management of the court with a turnaround strategy for the undertaking or to set up a turnaround plan for the undertakings.

Professional insolvency practitioners to manage a failed oil firm positively and to prevent a preventable liquidation where there are strong potentials that the oil firm remains sustainable and indebtedness could be due to improper governance, mismanagement, God's Act, force majeure and sudden adverse regulatory changes, among others, but these are not yet in place in Nigeria (Idigbe, 2017).

In Nigeria, under section 634, 635 of the Companies Allied Matters Act 2020, creditors may request the court to remove the veil of incorporation of the firm to make its directors personally liable if they are guilty of any crime or civil wrongdoing committed by an oil firm that they have been aware of. The firm will lose its privilege of legal persons, specifically where the directors who have signed the contract are personally liable. Sections 206, 211, 213 and 214 of the Insolvency Act of England and Wales have a provision similar to that which allows a liquidator or an administrator to sue the directors for unlawful trading knowing full well that the firm lacks the prospect of insolvency trading.

#### 3.3.2 Global experiences in insolvency and business recovery procedures

As the international fiscal upstream oil industry progresses, the core of contemporary indebtedness recovery practices and regulations has modified from the retribution of bankrupt oil firms via obligatory winding up to a new productive substitute, the reorganisation and rearrangement of such firms and their processes, to rehabilitate them to guarantee fiscal firmness and economic suitability. In Nigeria, though, the conventional method is maintained where the winding-up of the oil firm's venture and winding**-**up remains the sole mechanism for dealing with oil firm insolvency cases.

One of the current developments is that lawyers are now receiving more insolvency assignments than accountants. Banks, upstream oil firms and Assets Management Companies of Nigeria (AMCON) are now giving the highest insolvency assignments to Senior Advocates of Nigeria (SANs). The SANs would then hire the accountant as Financial Advisor to do so, with accountants now playing second place behind lawyers in insolvency and business recovery practices in the country. The Institute of Chartered Accountants of Nigeria (ICAN), being a specialised institution regulating the profession, must come to the rescue of accountants in this area.

Also, creditors, especially banks, now prefer the full realisation of charged assets rather than turnaround management. Since some lawyers are not trained as a business manager or manage a business, their approach to insolvency assignments is to take over charged assets and carry them out without considering turnaround options or a business rescued strategy.

The consequence of this is that jobs are lost in the event of a complete realisation of the assets charged while jobs are retained in the case of a turnaround management option. This is a far-reaching impact on the economy of the nation. This is the fundamental difference in insolvency practice in the relatively advanced economies and Nigeria.

In advanced economies, insolvency practice aims to reduce job losses while at the same time protecting creditors. Closing insolvent oil firms that can be successfully reverted has led to several job losses. A case in point is Afroil Plc's case. Some lawyers do not like turnaround management, but this is the area of competence of some Accountants.

Another current development is that the Corporate Affairs Commission (CAC) requires a copy of the Deed of Debenture to be submitted to the Receiver or Manager before his appointment is registered. This has not been the case until recently. In the past, the Corporate Affairs Commission (CAC) relied on the Deed of Debenture, which had previously been registered in the file of the insolvent oil firm.

Also, the Assets Management Companies of Nigeria (AMCON) Act tolerates the exercise of many arbitrary powers that are at variance with the investors' benefits and which can be utilised strictly against insolvent oil firms with minimum scrutiny. Some eminent legal scholars have proposed that specialised revenue courts should be established. This court has authority as the court of the first contact for the settlement of disputes in the upstream oil and financial sectors to curb the arbitrary nature of the current legal regime for dealing with insolvent oil firms in Nigeria.

Conversely, current insolvency regulations favoured substitution of balance due resolution procedures which would have saved insolvent firms rather than liquidated them. Generally, Nigeria's insolvency law appears to be deficient in terms of company improvement and reformation strategies. The new insolvency legal framework under the Companies Allied Matters Act (CAMA) 2020 provides for different classes of company insolvency procedures; non**-**collective proceedings (receipt) and collective proceedings (compensation, mergers and acquisitions) (Olisa Agbakoba and Associates 2015).

Another development in insolvency practice is the summary judgment procedure laid down in Order 13 of the Lagos High Court Rules of Civil Procedure 2019; this is another recovery mechanism used in Nigeria for the speedy recovery of debt. This procedure is only applicable in Lagos State. Summary judgment is a judgment that summarily favours the applicant without going through a full trial, in particular where the Defendant has no defence. In some instances, there may be no pleadings, but only an affidavit from the complainant and, if necessary, an affidavit from the plaintiff. The Defender's counter-affidavit. The proceedings shall be used where the applicant believes that the case's facts are straight forward and uncontested by the Defendant. It saves time and costs for lengthy trials, *Mcgregor Associates v. NNBN (1996) 2 SCNJ 72*.

However, a defendant who intends to defend the claim must do so within 42 days by filing within the limited time his defence Order 15 Rule 1(2), Lagos State Civil Procedure Rules, 2019. The prosecution must prove whether the Defendant denies the entire or part of the allegation and not just a general denial. In *Cotia Commercio E. Importacao SA v. Sanusi Brothers (Nig.)* Ltd. (2000) 6 SCNJ 453, the Supreme Court held that the mere general denial of a claim, a case of difficulty or inability to pay in the counter affidavit, or the filing of a frivolous defence, was not sufficient for the defence to be granted leave to the Defendant. Where the Defendant has made a prima facie argument, leave to appeal can be given. Order 13 Rule 1 Lagos State Civil Procedures. The 2019 case will then be included in the General Court File to be tried (Lagos State Civil Procedures Rules, 2019).

There is also a dispute resolution option for debt recovery; this is an informal dispute resolution mechanism where the individuals congregate with an expert who helps them settle their disagreement in a consensual and cheaper, not time-consuming compare to insolvency litigations. Other forms of alternative dispute resolution mechanisms include mediation, arbitration, and negotiation, among others.

The Alternative Dispute Resolution (ADR) is usually quicker Order 28 Rule 1–4. It is centred on direct participation by the parties to the case, rather than lawyers, judges and the judiciary. In most ADR procedures, the opposing parties specify the mechanism they will follow and describe the content of the negotiations. This type of participation is considered to enhance the people's happiness with the resolutions and their obedience with the agreements made. (Etigwe Uwa, Streamsowers & Kohn, 2017).

#### 3.3.3 Indications that an oil firm is under insolvency

Among other factors, the following are the symptoms to be found that an oil firm is close to insolvency, which mostly happens due to inadequate management: the lack of a formal investment strategy or a corporate development plan under which the oil firm plans to work within five years.

The absence of a strategic business plan limits the oil firm's chances of business success. The oil firm persistently refused to remit or often withholds deducted taxes to appropriate tax authorities to have access to cash to meet their immediate financial obligations. Lack of capacity to raise equity or loan capital In general, an oil firm with insufficient cash to pay its debts may raise capital by refinancing, raising equity or deferring debts, and inability to do so within a reasonable time indicate that such an oil firm is on the brink of insolvency (Andrew, and Walton, 2003).

Failure to provide timely and accurate financial information to the oil firm, where there is a persistent failure to provide financial information by the oil firm, may give rise to an inference that such an oil firm is insolvent. Also, where such a firm is generating incessant debits or an absence of adequate investment capital, this is a caution on the probability of insolvency and where the firm is persistently giving out post-dated cheques or where the banks reject its cheques. A post**-**dated check issued by an oil firm rejected in the absence of any irregularities is a clear sign that the firm is facing insolvency rather than temporary cash-flow difficulties (Gerard, and Klingebiel, 1997).

An oil firm is incapable of managing its cash flow properly, a profit and loss statement may often show that an oil firm is profitable, but the firm may be struggling to remain in business due to low cash flow. Its suppliers, consultants, are cutting off due to non-payment by such oil firm; this is a sign that such a firm is not managed correctly. Where there is a persistent experience of low sales, this is a clear sign of a dying oil firm, since sales are the vein of every oil firm and where the creditors of the firm issue notices of demands or legal notices. Proceedings for the recovery of outstanding debts, a single claim petition is not evidence of insolvency, since the debt may be contestable. Still, multiple appeals by several claimants may give rise to a presumption of insolvency among others.

### 3.4 Insolvent oil firms reformation approaches and other panaceas accessible to unsecured creditors

Generally, an unsecured creditor has no rights to insolvent oil firm assets unless he has obtained a court's judgment. Section 117 of the Investments and Securities Act LFN 2004 allows for a takeover bid strategy that provides for the purchase of appropriate stock in another oil firm (frequently denoted as the target oil firm) to grant the acquisition of an oil firm control over that other oil firm. It is distinct from a merger where the oil business has taken over the firm, but as a division of the purchasing oil firm. Thus, the oil firm's goals remain independent and distinct but as an affiliate of the purchasing oil firm. However, a firm cannot make a takeover bid either by another entity or on its own, until it has accepted the takeover offer of its managements, Section 139(1) of the Investments and Securities Act (Dentons, 2016).

It must be remembered, though, that there must be no fewer than 51% of the oil firm's stock to be bought for a merger acquisition. An offer under a takeover bid must be included in a record which must state or define the issue specified by section 136(1) of the Act. Shareholders of the offeree oil firm can approve or reject the offer presented to them regarding their shares.

Management-buy-out is another strategy; in this process, as the organisation performs a significant role. The transaction is carried out by a business party who uses the loan funding issued by a bank or other entity to purchase an insolvent oil firm.

The possessions of the oil firm so procured are covenanted as security for the credit facility, and the loan is expected to be repaid from the cash flow of the oil firm. The management buy-out can generate a conflict of interest if the management is a member of the buy-out party and may also represent the interests of the shareholders of the target oil firm.

There is also a transaction and assumption; there is a case where an oil producer chooses to buy from another oil firm, for example, when Oando Plc buys upstream oil business from ConocoPhillips, Nigeria. It is a complete purchasing and takeover of the other oil firm. Another tactic is hiring an administrator; an oil firm can join in the administration of an oil firm by joining an existing scheme to save the firm from insolvency. Insolvency professionals are in charge of the oil industry with a specified scheme to save the oil firm from insolvency.

Similarly, an oil producer's unsecured borrower may incorporate the protection of the title clause in the supply arrangement to shield it and ensure timely payment. Before the full purchase price is paid, the title of the products supplied may not be transferred to the purchaser. Likewise, an unsecured creditor may bring a suit for liquidation against an insolvent oil firm or may ask for an administrator's appointment after a range of written requests have been made. Still, the liquidation court has been used as a debt recovery mechanism in Nigeria.

**Figure 1:**
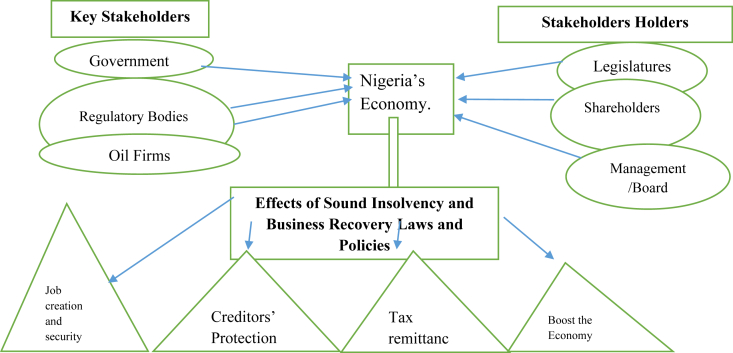
The Proposed Model of Nigeria's Insolvency and Business Recovery Legislations and Policies. Sources: The author prepared this.

There is the need for a model Nigeria's insolvency and business recovery legislations and policies to combat insolvency enhance fiscal stability in Nigeria's economy (see Figure 1).

#### 3.4.1 Liquidation of insolvent oil firm - as a final option

The winding-up of an insolvent oil firm may be carried out by the court, voluntarily by the owners (shareholders), by the creditors of the oil firm and subject to the oversight of the court in compliance with the terms of the section 714 Companies and Allied Matters Act, 2020 which should be the last resort after the collapse of all rescue and reconstruction procedures of the oil industry. Section 564 of the Companies and Allied Matters Act, 2020 provide the grounds for the insolvent oil firm's liquidation as follows: Where the firm has decided by special resolution that the firm should be liquidated, where the oil firm fails to submit statutory reports to the oil firm.

Corporate Affairs Commission (CAC) and where the number of shareholders of the oil firm has declined below two in the case of a firm with more than one shareholders, where the oil firm is unable to pay the number of its debts above 2,000 after several written submissions have been made to the oil firm and where the court thinks that it is reasonable and equal for such an oil firm to be liquidated as stated under section 572 Companies and Allied Matters Act 2020. If the oil film fails to hold the regulatory meeting and submits statutory reports.

An order can be made by the court, on the aggrieved individual's request, by the oil firm *suo motu*, to wind up by voluntary liquidation, as provided for in sections 620,622,626 of the CAMA, bypassing a special resolution to that effect in a general manner. Sections 625(1)(2)(a)(b)of the Act specifies, moreover, that directors shall file a formal statement of solvency to the extent that, after thorough study, they believed that such an oil firm would be in a position to pay its obligations in full within a duration not more than twelve months. Such a statement must be adopted within five weeks of the ratification of the resolution.

With the consent of its creditors, an oil firm may also decide to enter into voluntary liquidation. This is known as the voluntary winding-up of the creditor as provided for in Sections 714,715 and 716 of the CAMA. In a creditor's voluntary winding up, a discussion of the oil firm and the creditors for which sufficient notice has been submitted and reported once in at least (2) two newspapers and the federal gazette, is scheduled on the same day in accordance with sections 634, 635 of CAMA 2020. A full declaration of the status of the oil firm with the list of creditors and the total sum of their claims shall be made before the conference and a judgment on the firm's voluntary termination. At their meeting, the creditors shall engage a Board of Inspection comprising of not exceeding (5) five members to oversee the operation of the liquidation. The oil firm will also appoint an equal number to join the commission, but the creditors or the judge must accept any nomination.

The only situation in which an insolvent practitioner can expect maximum cooperation from the debtor is in the case of a voluntary termination by members, in all other cases, the insolvent practitioner should take an investigative attitude and consider any unusual friendliness of the debtor as a Greek gift until it has been proven otherwise. The monitoring of the debtor's assets may take on a significant dimension if the debtor happens to be one with an asset in other countries since the aggregation or discovery of those assets may require the application of foreign laws and may also depend on whether Nigeria has diplomatic ties with the country concerned.

Whatever action the practitioner takes to track the asset, he should bear in mind that such action must be cost-effective. He must increase the dividend payable to creditors without unduly delaying the assignment. Immediately the winding-up order shall be issued by the judge, the official receiver by his office shall become the temporary liquidator if none has been named and, after the nomination of the official receiver, the functions of the boards shall end - section 579 CAMA. No legal action shall be taken or re-opened against the oil firm except with the court's leave: Section 417, CAMA. Any sale of the oil firm's assets, the transmission of its stocks or modification of the position of its memberships after the beginning of the liquidation actions shall be null and void except ordered by the court. Any supplement, appropriation, concern, or implementation against the assets or the oil firm's consequences is invalid at the start of the winding-up. After the winding-up order has been released, the oil firm's activities will be terminated.

The winding-up proceeding should be the final resort or not to be used by the Nigerian firm for debt recovery. *Eastern* *Airlines Limited .v. Air via Ltd. (1998)* 12 NWLR *(Pt. 577) 271 at 280–281, Hansa International Construction Limited v. Mobil Producing Nigeria (1994)* 9 NWLR *(Pt. 336) 76 at 86, Nigeria Industrial Development Bank Ltd. v. Fembo Nigeria Limited (1997) 2 FHCLR 501 at 502.* Alternatively, unsecured creditors may order the release of the insolvent oil firm's shares, the formation of the initial payment or the *pari passu* fee.

The oil firm may change its share capital by a recapitalisation. An oil firm can also opt for a scheme of agreement or compromise by amending the form of rights and preference shares to pay the accumulated unpaid dividends. Unsecured creditors can take up shares in an oil firm or take part in cash to pay the debt owed. Creditors may ask the court for the judicial sale of the oil firm's assets to meet the outstanding debts and the commencement of legal proceedings for the repossession of the principal financial obligation and the interest if any.

The creditors may order the court to withdraw the cloak of incorporation of the oil firm and make its directors legally liable if they are guilty of any crime or civil wrongs committed by the oil firm, in particular, if they have signed personal guarantee contracts and are aware of the firm's financial inability and trade.

Similarly, Sections 206, 211, 213 and 214 of the Insolvency Act of England and Wales have a clause similar to that which enables the liquidator or the receiver to sue the directors for unfair trading full well that the firm lacks trade opportunities.

Any deal carried out with the loan may be rescinded by the oil firm where there is an aspect of deception, misrepresentation and penalties can also be sought. The Director may be suspended for fifteen years under the United Kingdom Firm Directors Disqualification Act, 1986. The payments to creditors are made in the following arrangement of significance: Creditors safeguarded by fixed charge, preferential creditors, creditors fortified by floating charge, protected but contractually subordinated creditors and lastly the unsecured creditors (Latham and Watkins 2017).

#### 3.4.2 What lessons can Nigeria absorb from the designated case study nations?

There are also lessons to be learned from the jurisdictions' solvency and debt recovery law system under review that serves as a blueprint for Nigeria's solvency and debt recovery legal framework reform. Recent restructuring trends in other countries, such as Malaysia, India and South Africa, among others (Khan et al., 2014).

Firm consolidation and stabilisation plans include a restatement of the insolvent oil firms' debts and obligations and conducting negotiations with banks to make provisions for repayments of the outstanding loans. The reorganisation is an effort to lengthen the existence of an oil firm tackling insolvency via a distinct restructuring to minimise the option of previous insolvency circumstances re-occurring. Oil companies can renegotiate their debts with their creditors to get better terms, and the firm can continue operating and works toward repaying its outstanding debts.

Compromise or agreement under the Companies Allied Acts, Cap C.20 LFN, 2004 and the England and Wales Companies Act, 2006 creditors and owners can be used for the internal resolution of an insolvent oil firm. This is an agreement under which an oil firm with the creditors and the owners consider less than what they are eligible to fulfil the firm's contractual duty to them (Olujobi, 2020a).

Compromise occurs when the oil firm persuades its creditors to take stock or half of the stock and half of the cash to cover its obligations as the transaction is a method of acquisition.

Controlling shares or transferring an oil trade or part of it to alternative an oil firm is considered for stocks. It is important to note that another private oil firm cannot take over a private oil firm, but another private oil firm can do the same. On the other hand, the holders of preference shares may be compelled by the insolvent oil firm to cancel the accumulated returns on investments, decrease the fixed amount of the dividend, or allow the transfer of their preferred stocks to common shares to minimise the number of assets (Bernard, 2015).

A compromise or arrangement under sections 710–711 of Companies Allied Matters, 2020 and the England and Wales Companies Act, 2006 creditors and shareholders may be used to internalise an insolvent oil firm. This is an arrangement by which an oil firm with its creditors and stockholders accepts less than they are allowed as the fulfilment of the financial responsibilities of the firm to them. Also, an arrangement on sale is provided for in Section 538 of the CAMA; an oil firm may decide by special resolution that the oil firm should be terminated and a receiver may be chosen to vend all or portion of the assets of the firm to another oil firm for cash, shares or debts as a consideration which the receiver shall allocate respectively to the investors in conformity with their liquidation claims (Wihlborg Clas, 2002).

An arrangement under Sections 434–442 of the Companies Allied Matters, Act 2020 and the England and Wales Companies Act, 2006 creditors and shareholders may be used to internalise the insolvent oil firms. This is an agreement whereby an oil firm with the creditors and shareholders, recognises below what they are eligible to as the fulfilment of the contractual duty of the firm towards them. Notice that any shareholder may demand that he or she is not a party to the agreement and that no opposing shareholder will be allowed to take an interest in the oil firm since he or she has expressed his or her opposition under the statute.

**Figure 2:**
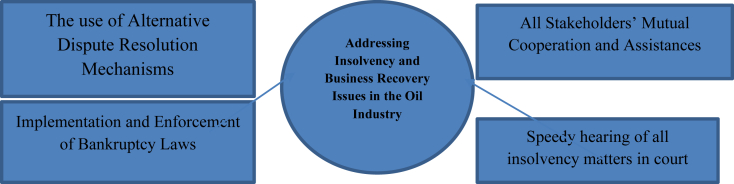
Hybrid Methods of Addressing Insolvency and Business Recovery Issues in the Oil Industry. Sources: The authors prepared this.

**Figure 3:**
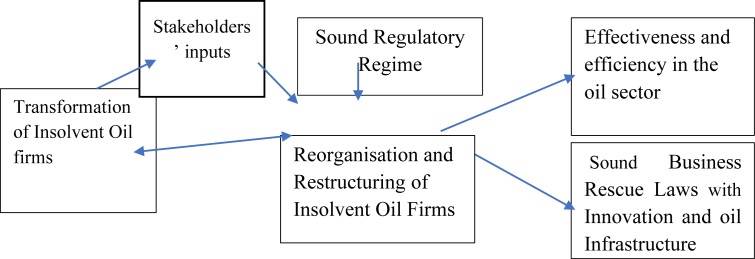
Benefits of Sound Insolvency and Business Recovery Laws Model in the Oil Industry. Sources: The author prepared this.

Hybrid methods of combating insolvency and business recovery legal issues in the oil industry are highlighted below (see Figure 2):

It is pertinent to note that proficient, dependable, and translucent creditors and debtors' legal regimes and insolvency arrangements are fundamental to Nigeria's government for speedy economic recovery from the adverse global effects of the Covid-19 pandemic (see Figure 3).

The paper has contributed to knowledge by providing insight on how insolvency laws and regulatory institutions can drive the needed reforms in the oil industry. The study is valuable to Nigeria's being an emerging economy. Besides, the study offers a model for combating insolvency in the oil industry. Since there is no evidence that a single study has earlier identified the challenges of insolvency and business recovery issues in the oil industry and compares them with experiences in other jurisdictions and compliance with international standards, this study is beneficial.

## 4 Discussions of findings

Nigeria's regulatory process for reforming insolvent oil firm is insufficient. The statutory insolvency legal framework appears to be leaning towards liquidation proceedings. The idea of corporate rescue is not new in Nigeria. Some of these provisions stated under sections 184 (1),186, 710,711 the Companies and Allied Matters Act, 2020 such as agreements, concessions, takeovers, amalgamations/acquisitions under the Investments and Securities Act, 2007. However, our existing rescue laws are not well organised as the traditional western business rescue laws, they are inconsistent with current global best practices, they ignore restructuring processes for insolvent oil firms, and they even fail to allow for a comprehensive process for licensing of insolvent and business recovery practitioners in Nigeria.

Sections 658 and 659 of CAMA 2020 fail to define a connected person, and section 658(6) fails to clearly define appropriate time which ought to be two years after the commencement of insolvency. This is similar to sections 238, 239 and 240(2) of the United Kingdom Insolvency Act 1986 which describe an appropriate time to consider the concept of fraudulent preference and undervalued contracts to be two years.

The numerous difficulties facing business rescue options these difficulties point to the need for well-structured business rescue laws that will assist oil firms in Nigeria, whether by harmonised insolvency law or piecemeal changes in our existing insolvency legislations. It was also observed that heavy reliance on the court system makes the rescue of the insolvent oil firm more cumbersome, which may not be cost-effective and time-effective for oil firms. South Africa restructured the agreement and settlement clauses of its Companies Act, 2008 to exclude one step of court action: the court-ordered meeting that Nigeria should be replicated for successful oil firm business rescue to guarantee efficiency and good governance.

In addition to enacting a coherent legal system, the legislation requires clarity. The provisions of any harmonised legal framework must be stated clearly, and in plain words, those who have to apply it can readily understand. Lack of clarification is disruptive to the law and impertinent to those seeking to uphold the rule of law and enabling those seeking to abolish it.

Therefore, there is the need to build Nigeria's business rescue legal structure, such as the selected case study countries, which is debtor recovery-oriented generally known as a business rescue. This alternative means that insolvent oil firms are spared from liquidation, and only those oil firms with little hope of surviving are liquidated in light of the critical positions that oil industry plays in many developed countries economies, resulting in many countries reforming their financial structures and improving their insolvency legislations through corporate rescue and reconstruction.

For Nigeria's upstream petroleum industry to improve insolvency and firm rehabilitation procedures, there is a need for a comprehensive and committed insolvency law or system that would cover all forms of insolvencies. Even in the United Kingdom, where the two laws remain distinct, a specific bankruptcy statute remains. What is currently applicable is the Insolvency Act of 1986, which crosses the distance between the rules and procedures regulating individuals' and energy industry insolvency.

The way forward for us could be to provide business rescue schemes that allow the debtor to remain in control, maybe under the rescue practitioners’ oversight to ensure checks and balances. The method of making the same rescue specialist the liquidator of the oil firm where the rescue collapsed is a big pitfall to prevent. The goals of the rescue professional and the liquidator are contradictory. If we followed this approach; the rescue specialist would, of course, continue to receive payments. As a result, no reasonable effort will be made to rescue the insolvent oil firm because the rescue practitioner earns more if the oil business fails.

The value of providing a well-structured market rescue system cannot be over emphasised. The advantages to the debtors and borrowers and society as a whole are numerous. Employment is sustained, oil firms are more likely to take commercial chances, foreign investment is welcomed to invest in the oil industry, and Nigeria's economy will be boosted.

Therefore, we need workable rescue mechanisms that can be easily enforced. At the minimum cost with fundamental issues such as critical indicators for initiating business rescue, especially for managers, versatility in terms of both formal and informal approaches, moratorium requirements, uniform qualifications for rescue practitioners, little court involvement, provision for rescue oil firms' employee contract, recognised the insurance or trust to be issued by rescue practitioners against criminal sanctions. These laws are not the only ones aligned with current global developments but would ensure that adequate rescue and oil-business friendly policies are enacted in Nigeria (Olujobi and Oyewunmi, 2017).

### 4.1 Recommendations

It is trite that an efficient insolvency legal regime will facilitate the rehabilitation of indebted oil firms and offer a useful tool for the winding up of oil firms that cannot be transformed (Hagan, 2000) As the world is increasingly heading towards a firm rescue system that is welcoming, open to debtor oil firms which can be extended to all businesses like the United States’ model that South Africa recently introduced with some modifications. In recognition of the paradigm change, even the United Kingdom had already made many changes to their legal rules for business rescue by significantly reducing the effectiveness of the receivership and changing the business rescue mechanism directed by their courts. Whereas the regulations relating to firm recovery, i.e. firm voluntary agreement and receivership may be useful in selected case study countries that have been explicitly tailored to suit their jurisdictional needs to avert lack of interaction between the legislation and the Nigerians. In deciding the best oil firm recovery and turnaround model for Nigeria, the following points are noteworthy:

First is to understand the extent of the court's intervention and what step of the rescue procedure would entail the court. Our courts are now overburdened with cases; thus rescue solutions that are very scalable with limited court control would be more fitting for Nigerian oil businesses.

It is also essential not to force new management on the oil firm in the form of a rescue practitioner who might not even know anything about the oil industry. In realistic terms, those who support the change of management will end up placing the rescue specialist in a role where they will have to employ the professional service in order to be able to save the oil firm and from incurring potential costs. Where managers or administrators are eliminated and replaced by rescue personnel, then many problems occur.

Second, the rescue specialist is received with hostility; he gets little to no support from the administration and considers the rescue goals quite challenging. Because of our existing receivership practice in Nigeria, these lessons are not farfetched. To take over the insolvent oil firm while the Nigerian receiver will need the support of law enforcement officials and, in most situations, the business will face significant opposition.

The problem of debt recovery through the insolvency proceedings by an oil firm in Nigeria devoid of litigation is through Alternative Dispute Resolution (ADR) mechanisms by settling out of court. It decreases the budget for litigation and prolonged delays associated with legal actions. The firm is wound-up, and liquidators are named, the debtors will not reclaim the entire debts due, thus incurring further costs and losses on the firm that they hope to save for their shareholders.

Therefore, a successful insolvency law must be in a position to remove bankrupt oil firms and create trust in the country's economy. In addition to defining the fundamental elements of insolvencies or bankruptcies, the legal system should create special courts dealing with bankruptcies and insolvency matters. The selection of judges, who are commercial law experts to sit on insolvency matters, is essential. The special courts to be established should be limited to handling insolvency lawsuits and unsecured creditors, allowing protected creditors to continue finding redress from the traditional courts. Since the court is the last resort, litigation can only be taken against those oil firm debtors who, considering their willingness to do so, cannot comply in paying their debts and against the debtors who are behaving in lousy conscience in paying their debts.

The author recommends that the Federal Government passed a regulation forcing financial firms to help insolvent oil firms restructure their debt and allow insolvent oil firms to stay in office with insolvent and business recovery practitioners supervising their activities enactment Code of practice for insolvency practitioners in the country.

There is a need to amend the Companies Regulations and the Companies Procedure Rules in conformity with the new legal regime on corporate law in Nigeria. Resilient regulatory institutions and laws are vital to an efficient insolvency system.

#### 4.1.1 Study limitations/implications

The research findings could be lacking in generalizability because of the research methodology chosen. Future scholars are also urged to use quantitative analysis methodology to evaluate the suggested propositions further to further enriches the existing literature on the subject.

## 5 Conclusions and policy implications

The efficient legal system for enforcing debt claims by oil firm is fundamental to secured and unsecured creditors for magnificent yields to creditors and restructuring of sustainable oil firms.

Having analysed the theoretical structure on insolvency and business recovery practices in Nigeria with a comparative study of Nigeria, Malaysia, India, and South Africa's current legal framework on insolvency, the Nigerian legal system's biggest weakness is the lack of committed, comprehensive and integrated regulatory structure for insolvency practice.

The author's opinion that analysis of the existing insolvency and rehabilitation laws shows some unanswered issues that have been a source of concern for insolvent practitioners. For instance, the Bankruptcy Act in Nigeria has not been amended since 1992 with other obsolete insolvency rules, Bankruptcy Statute and the Dishonoured Cheques Clause, need to be amended. The Nigerian insolvency legislation should be updated to include a more impartial mechanism for safeguarding debtors and borrowers alike and now is the best time to do so. We must not wait for a big financial crisis to arise until we embark on legislative reforms.

Despite the shortcomings of the current insolvency system, the courts have a crucial part in administering administration justice and managing the needs of the different parties in insolvency**-**based contractual conflicts. The courts will strategically create a paradigm change in our insolvency system for business rescue and insolvency practice in Nigeria through effective adjudicatory power. The sustainability of oil firms may be improved by adjusting their strategies to manage prices, track revenues, and assess potential market prospects' feasibility in the industry (Olujobi, 2020b). Sections 658 and 659 CAMA 2020 will aid administrators or Insolvent practitioners on recuperating insolvent oil firms' assets tracing, which may be difficult before now in Nigeria.

The management of some oil firms who often sell and conceals firm's assets on the belief of imminent insolvency proceedings and who often dole out the firm's assets as gifts to their relations should conscious that such actions may be set aside and assets salvaged if such firms are placed under an administrator or liquidator (Chiwete, 2020).

If compiled, the recommendations will have a positive impact on insolvent oil firms in financial difficulties to continue doing business while undergoing significant reforms. Finally, introducing a voluntary corporate arrangement under the insolvency legal regime as entrenched in the Companies and Allied Matters Act 2020 will allow an oil firm to settle its financial obligations installmentally.

This is through an arrangement with its creditors by continuing in business, thereby opening doors for easy business ways. However, there is a need for improvement in the Nigerian legal landscape to do businesses in the oil industry, both locally and globally, to attract more foreign investors to the industry.

## Declarations

### Author contribution statement

Olujobi, Olusola Joshua: Conceived and designed the analysis; Analyzed and interpreted the data; Contributed analysis tools or data; Wrote the paper.

### Funding statement

This research did not receive any specific grant from funding agencies in the public, commercial, or not-for-profit sectors.

### Data availability statement

No data was used for the research described in the article.

### Declaration of interests statement

The authors declare no conflict of interest.

### Additional information

No additional information is available for this paper.
